# The current status of early nursing for emergency pancreatitis and analysis of factors influencing prognosis: A retrospective study

**DOI:** 10.1097/MD.0000000000039662

**Published:** 2024-09-27

**Authors:** Lejuan Xu, Fengxin Li, Jiehua Wu, Liang He, Zhe Gao

**Affiliations:** a Department of Emergency, The Second Affiliated Hospital, Jiangxi Medical College, Nanchang University, Nanchang, Jiangxi, China.

**Keywords:** early care, experimental indicators, pain situation, pancreatitis, prognostic impact

## Abstract

This study is to investigate the incidence of pain and the influencing factors of prognostic complications in early admission care of pancreatitis in the emergency department patients. This provides a basis for clinical nursing management and prognosis improvement. Hundred patients with acute pancreatitis admitted to the tertiary hospital between January 2021 and December 2023 were selected according to the inclusion and exclusion criteria. It collected basic baseline data and medical data of patients during admission, clarifies whether patients have complications, and analyzed the length of hospital stay. Comparing hospital stays >7 days with <7 days. A questionnaire on patient psychological status was collected, and single factor analysis was conducted on different prognostic factors. Binary logistic regression was used for single factor analysis, and *P* < .05 was considered statistically significant. The presence or absence of complications during treatment is the main criterion for determining the prognostic impact of pancreatitis in the emergency department patients. Among 100 patients, 26 (26%) had complications during hospitalization, 74 (74%) had no complications during hospitalization, and 64 (64%) had a stay of >7 days. There were statistically significant differences (*P* < .05) in smoking status and history of hypertension between the complication group and the non-complication group. In the comparison between the group with <7 days of hospitalization and the group with >7 days of hospitalization, age, education level, smoking status, and history of hyperlipidemia showed statistical significance (*P* < .05). The fasting days, BISAP score, first bowel movement time, C-reactive protein, blood urea nitrogen, albumin, duration of severe pain within 24 hours of admission, and duration of severe pain within 24 to 48 hours of admission were all statistically significant (*P* < .05). Pancreatitis in the emergency department patients are prone to exacerbation and prolonged pain during early hospitalization. In nursing, timely attention should be paid to the patient’s pain issues, timely pain relief measures should be taken, and the occurrence of complications should be reduced, reducing the patient’s hospitalization time. Meanwhile, it is necessary to constantly pay attention to changes in the patient’s gastrointestinal function and experimental indicators.

## 1. Introduction

Acute pancreatitis (AP), as a common acute abdominal disease, has shown a significant upward trend in incidence in recent years.^[1]^ The clinical manifestations of AP are diverse, and the condition progresses rapidly, which can lead to various complications, and in severe cases, even endanger life.^[2]^ Due to the complexity and urgency of AP, early and accurate diagnosis and timely and effective care are crucial for improving patient prognosis. In recent years, with the advancement of medical technology and the deepening of nursing practice, there has been a deeper research and understanding of early nursing methods and prognostic factors for AP.^[3]^ However, despite multiple studies focusing on drug therapy and surgical intervention, further exploration is needed on the specific practices and prognostic factors of early care for AP. Early care not only includes basic care such as pain control, nutritional support, and monitoring of patient vital signs, but also involves psychological support and health education. These all have a significant impact on improving the quality of life of patients and reducing the occurrence of complications.^[4]^ This study analyzes the early admission care and pain occurrence of pancreatitis in the emergency department patients, and explores the pain occurrence and pain relief measures in the current early admission care of pancreatitis patients. This has analyzed and explored the multifaceted impact on the prognosis of patients. This provides a new direction for further clarifying the early pain situation and prognostic factors of pancreatitis in the emergency department patients, as well as new clinical indicators for reducing hospital stay and early care.

## 2. Data and research methods

### 2.1. Research object

This study was approved by The Ethics Committee of The second affiliated Hospital. Selecting 100 patients with AP admitted to tertiary hospital from January 2021 to December 2023 as the research subjects. On the grounds of the patient’s condition during treatment, 100 patients were divided into groups with and without complications. The inclusion and exclusion criteria for patients are as follows:

Inclusion criteria: (1) patients are all adults and must be no <18 years old. (2) Select the diagnostic criteria for AP in the Chinese Guidelines for the Diagnosis and Treatment of AP, which meet the abdominal pain symptoms of AP.^[5]^ Examination shows a significant increase in serum amylase and lipase. Imaging findings include pancreatic edema, peripancreatic exudation, and pancreatic and/or peripancreatic tissue necrosis. (3) The patient received conservative treatment during hospitalization. (4) The patient’s consciousness is clear, their language expression is unobstructed, and their mental state is normal.^[6]^ (5) The patient has no barriers to understanding, clear logic, and is able to express their own situation normally. (6) Patients who have received informed consent from their family and are willing to participate in the study.

Exclusion criteria: (1) corresponding pain treatment has been received. (2) There are other diseases that cause pain and cannot cooperate with the experiment. (3) Patients undergoing surgical procedures during treatment.^[7]^ (4) Cognitive impairment, mental disorders, inability to engage in language communication.^[8]^ (5) Unable to complete the research in its entirety.

### 2.2. General baseline data

The general patient data standards used in this study are age, gender, and body mass index. The calculation is on the grounds of the measurement of height and weight during the patient’s pain relief or pain relief period, calculated according to the standards of the World Health Organization.^[9]^ Smoking status includes classification of frequent and occasional smoking, as well as smoking cessation status, alcohol consumption status including frequency of alcohol consumption, and whether alcohol has been quit. Is there a history of recurrence in AP. Past medical history includes a history of hypertension, hyperlipidemia, and fatty liver. Smoking status includes classification of frequent and occasional smoking, while quitting smoking indicates that the patient has stopped smoking for 2 consecutive years. Drinking status refers to the frequency of drinking alcoholic beverages per week in the past year, while quitting alcohol indicates that the patient has stopped drinking for a consecutive year. Recurrent cases refer to cases with a history of 2 or more episodes of AP, and the interval between the 2 episodes is at least 3 months.^[10]^ A past medical history is on the grounds of the relevant diseases that were clearly diagnosed by the clinical physician in the patient’s previous medical records. The investigator needs to complete the survey of these general information within 48 hours of the patient’s admission.

The pain questionnaire for patients with AP is designed to evaluate the pain indicators of current patients on the grounds of existing scales. The pain assessment results are divided on the grounds of the patient’s mild pain, duration, and pain situation within 48 hours of admission. Using the NRS digital scoring scale, the pain index is divided into 0 to 10 points, where 0 points indicate no current pain, 1 to 3 points indicate mild current pain, 4 to 6 points indicate moderate current pain, and 7 to 10 points indicate severe current pain. The duration of pain is expressed by the patient themselves. The patient’s data and pain expression should be collected within 48 hours before the patient’s admission for treatment.^[11]^

### 2.3. Patient medical data collection

The medical information of the patient with AP includes biliary pancreatitis: bile duct stones, inflammation, cysts, polyps, biliary diverticula or roundworm infection, as well as confirmed dilation of the common bile duct through imaging detection. Hyperlipidemia associated pancreatitis: when the serum triglyceride level exceeds 5.6 mmol/L and other causes are excluded. Alcohol induced pancreatitis: the patient has a clear history of alcohol intake, such as drinking >50 g of alcohol per day for 5 consecutive years, while excluding other causes.^[12]^ Primary pancreatitis: no biliary related diseases were found, the patient had no history of excessive alcohol consumption, and no other causes were found through biochemical and imaging examinations. AP with mixed causes: diagnosed as AP containing at least 2 different etiologies. These causes are determined through medical history inquiries and diagnosis by clinical doctors.^[13]^ The experimental measurement indicators include neutrophil-to-lymphocyte ratio (NLR), glucose (Glu), C-reactive protein (CRP), calcium, blood urea nitrogen (BUN), albumin (ALB), and total bilirubin values, which were collected from the hospital’s electronic medical record system within 48 hours of patient admission. If there are multiple identical test results, the study selects the one with the worst result. BISAP scoring system: it is used to evaluate the severity of the condition in patients with AP. Within 48 hours after admission, the patient was scored on the grounds of the following 5 indicators: BUN value, state of consciousness, occurrence of systemic inflammatory response syndrome (SIRS), age, and pleural effusion. For each issue, 1 point is awarded, with a maximum score of 5 points. If the total score reaches or exceeds 3 points, moderate to severe or severe AP should be considered. Among them, the definition of SIRS includes at least 2 of the following signs: abnormal body temperature, rapid heart rate, frequent breathing or abnormal blood gas analysis, abnormal white blood cell count or increased immature cells.

### 2.4. Patient prognosis survey form

The patient prognosis survey mainly includes the collection of survey information on the patient’s comorbidities, mortality, and hospitalization status. According to the Chinese Guidelines for the Diagnosis and Treatment of AP (2021), the prognosis and complications of patients were evaluated. Systemic complications mainly include SIRS, sepsis, multiple organ dysfunction syndrome, abdominal hypertension, and abdominal compartment syndrome.^[14]^ Local complications are related to the accumulation of fluid and tissue necrosis in the pancreas and its surrounding areas. This includes but is not limited to early (<4 weeks) acute peripancreatic fluid accumulation, acute necrotic accumulation, and later (>4 weeks) pancreatic pseudocysts and encapsulated necrosis. Other complications include gastrointestinal bleeding, abdominal bleeding, intestinal obstruction, intestinal fistula, etc. In this study, complications were defined as 1 or more of the aforementioned complications that occurred during hospitalization. Death refers to the death that occurs during hospitalization due to AP. Hospitalization time refers to the total number of days a patient has received treatment in the hospital. According to the guidelines, a hospital stay of 7 days or more is considered an extension of hospital stay.

### 2.5. Data collection methods

According to relevant standards and literature guidelines, patients should undergo pain assessment upon admission and it is recommended to assess pain once a day. The evaluation period and purpose are to reduce memory bias in patients when recalling pain. Pain assessment is divided into 4 stages: early admission (0–2 hours), early post admission (2–24 hours), mid admission (24–48 hours), and post discharge. In the early stage of admission (0–2 hours), the main assessment is the patient’s pain condition before receiving treatment. The early and middle stages (2–24 hours and 24–48 hours) after admission mainly focus on the patient’s pain changes after receiving treatment. Specific evaluation execution plan: (1) first evaluation: conducted within 0 to 2 hours after the patient’s admission. (2) Second evaluation: conducted within 2 to 24 hours after patient admission. (3) Third evaluation: conducted within 24 to 48 hours after the patient’s admission, and during this period, collect patient pain data, general information, medical information (including etiology, laboratory indicators, BISAP score, gastrointestinal function status), as well as psychological and social information. (4) After discharge: collect and organize patient prognosis data.

### 2.6. Ethic principles

(1) Ethical approval: this study has been approved by the ethics committee of the hospital where it is located. (2) Participants agree that this study was conducted on a voluntary basis. All participating patients and their families have been fully informed of the details of the study, and have expressed understanding and voluntarily joined the study. (3) Privacy protection: to protect patient privacy, all medical data will be labeled with a dedicated study number instead of using the patient’s name. The research team will strictly keep all collected information and data confidential and only use them for scientific research purposes.

### 2.7. Data processing methods

Data entry and statistical software: the collected data is double entered using Excel spreadsheets by 2 independent staff members to ensure data accuracy. When conducting statistical analysis on data, IBM SPSS Statistics 24.0 software is used. The statistically significant difference (SD) standard is set to *P* ≤ .05.^[15]^ Statistical method: descriptive statistical methods are used for general baseline data, pain status, disease information, scoring, and disease prognosis information. For quantitative data that follows a normal distribution, the mean and standard deviation are used to represent it. For non-normally distributed data, median, and quartile ranges are used for representation. Qualitative data is described through frequency, proportion, and rate. Univariate analysis: when comparing quantitative data between 2 data groups, if the data group follows a normal distribution, use 2 independent sample *t* tests. If it does not follow a normal distribution, nonparametric tests will be used. The chi square test is used for inter group comparison of qualitative data.^[16]^ Multivariate analysis: it conducts binary logistic regression model analysis on factors that may affect the occurrence of complications and prolonged hospital stay, and screens variables from statistically significant indicators in univariate analysis. A bilateral test was used in the analysis, with *P* < .05 as the statistically significant criterion.

## 3. Result

### 3.1. General baseline data of patients

In this study, a total of 100 patients with AP were investigated, and 45 patients were diagnosed with biliary disease. The cause of the disease belongs to 29 patients with hyperlipidemia. The cause of the disease belongs to 3 alcoholic patients. There are 17 patients with idiopathic causes of the disease. There are 6 patients with mixed causes of illness, 63 patients diagnosed with severe pancreatitis, 19 patients diagnosed with moderate pancreatitis, and 18 patients diagnosed with mild pancreatitis. The specific investigation situation is shown in Table 1.

**Table 1:** Comparison of patients’ general baseline data.

Project	Classification	Number of cases (example)	Constituent ratio (%)
Age (years)	<60	72	72
	≥60	28	28
Gender	Male	61	61
	Female	39	39
BMI (kg/m^2^)	<18.5	7	7
	18.5–23.9	42	42
	24–27.9	43	43
	≥28	8	8
Fatty liver	Yes	46	46
	No	54	54
Disease severity	Mild symptoms	63	63
	Moderately severe	19	19
	Severe	18	18
Smoking status	Smoke	21	21
	Not smoking or quitting smoking	79	79
Alcohol consumption status	Drink	32	32
	Not drinking or abstaining from alcohol	68	68
Is this a recurrence	Yes	27	27
	No	73	73
The cause of this illness	Biliary	45	45
	High fat content	29	29
	Alcoholic	3	3
	Idiopathic	17	17
	Mixed etiology	6	6
Hypertension	Yes	35	35
	No	65	65
Hyperlipidemia	Yes	36	36
	No	64	64

BMI = body mass index.

### 3.2. Duration of different pain levels in patients with pancreatitis upon admission

As shown in Table 1, the longest duration of mild to moderate pain within 24 hours of patient admission is 24 hours. The longest duration of severe pain is also 24 hours. Within 24 to 48 hours of admission: The longest duration of mild to moderate pain remains 24 hours. The longest duration of severe pain is reduced to 18 hours, as shown in Table 2.

**Table 2:** Duration of different pain levels in patients with pancreatitis upon admission.

Project	Classification	*M* (*P*25, *P*75)
Pain relief situation	Pain relief within 24 hours [n (%)]	58 (58)
	No pain relief within 24 hours [n (%)]	42 (42)
	Pain relief within 24–48 hours [n (%)]	18 (18)
	No pain relief after 24–48 hours [n (%)]	82 (82)
Analgesic frequency (times)	/	1 (0,2)
Total accumulated time of pain during treatment (days)	/	3 (2,5)
Pain scale	Duration of mild to moderate pain within 24 hours (h)	11 (11,19)
	Severe pain duration within 24 hours (h)	4 (2,6)
	24–48 hours of mild to moderate pain duration (h)	5 (5,20)
	24–48 hours of severe pain duration (h)	0 (0,1)
	48 hours of total pain time (h)	6 (6,36)

### 3.3. Prognostic data of patients with pancreatitis

As shown in Table 3, a total of 27 patients experienced local or systemic complications. Among the local complications, 19 patients experienced local complications. Among them, 9 cases experienced APFC, 8 cases experienced acute necrotic accumulation, 2 cases developed pancreatic pseudocysts, and 1 case developed encapsulated necrosis. Among the systemic complications, 30 patients experienced systemic complications. Among them, 26 cases developed SIRS, 3 cases developed sepsis, 3 cases developed acute respiratory distress syndrome, 11 cases developed organ failure, and 2 cases developed intra-abdominal hypertension or compartment syndrome. Among the hospital stay and mortality rates, 64 patients experienced prolonged hospital stay, and 2 patients died due to worsening of their condition and varying degrees of organ failure.

**Table 3:** Prognostic diagnostic data of patients with pancreatitis.

Project	Classification	Number of cases (example)	Composition ratio (%)
Complication	Yes	26	26
	No	74	74
Hospitalization days	<7 days	36	36
	≥7days	64	64
Death	Yes	2	2
	No	98	98

### 3.4. Univariate analysis of general baseline data in early admission of patients with pancreatitis

As shown in Table 4, the results indicate that there is a statistically SD (*P* < .05) between smoking status and history of fatty liver disease in the occurrence of complications in patients with AP.

**Table 4:** Univariate analysis of general baseline data in early admission of patients with pancreatitis.

Project	Classification	No complications (n = 73) [n(%)]	There are complications (n = 27) [n(%)]	χ^2^	*P*
Fatty liver	Yes	33 (33)	20 (20)	4.623	.015
	No	67 (67)	80 (80)	/	/
Disease severity	Mild symptoms	32 (32)	32 (32)	0.001	.843
	Moderately severe	68 (68)	68 (68)	/	/
	Severe	28 (28)	27 (27)	0.009	.876
Smoking Status	Smoke	33 (33)	20 (20)	/	/
	Not smoking or quitting smoking	30 (30)	46 (46)	6.231	.007
Alcohol consumption status	Drink	70 (70)	54 (54)		
	Not drinking or abstaining from alcohol	35 (35)	40 (40)	0.463	.321
Is this a recurrence	Yes	65 (65)	61 (61)	/	/
	No	44 (44)	54 (54)	2.187	.095
The cause of this illness	Biliary	56 (56)	46 (46)	/	/
	High fat content	43 (43)	52 (52)	5.836	.176
	Alcoholic	30 (30)	28 (28)	/	/
	Idiopathic	4 (4)	2 (2)	/	/
	Mixed etiology	20 (20)	11 (11)	/	/

### 3.5. Comparison of complications in clinical data of patients with pancreatitis

As shown in Table 5, in the comparison of clinical data and complications in patients with pancreatitis, fasting days, BISAP score, patient’s first bowel movement time, NLR, Glu, CRP, ALB, and BUN all have an impact on the occurrence of complications in patients with pancreatitis. The difference possessed statistical significance (SS) (*P* < .05).

**Table 5:** Comparison of complications in clinical data of pancreatitis patients.

Project	No complications (n = 73) *M* (*P*25,*P*75)	Complications present (n = 27) *M* (*P*25,*P*75)	*Z*	*P*
Fasting days (days)	3 (2,4)	5 (5,8)	‐8.956	<.001
BISAP score (points)	1 (0,1)	1 (2,4)	‐9.354	<.001
First bowel movement time (days)	1 (1,2)	2 (3,5)	‐6.231	<.001
NLR (%)	7 (4, 11)	13 (9, 21)	‐4.356	<.001
Glu (mmol/L)	27.83 (2.89, 83.56)	162.46 (89.98, 219.86)	‐7.968	<.001
CRP (mg/L)	1.98 (1.946.25)	1.84 (1.76, 2.21)	‐6.657	<.001
BUN (mmol/L)	4.98 (3.95, 6.32)	5.987 (4.24, 9.95)	‐3.458	.01
ALB (g/L)	43.32 (39.02, 46.23)	33.98 (30.02, 38.15)	‐8.765	<.001
TBIL (μmol/L)	22.95 (13.86, 36.12)	23.94 (18.23, 38.02)	‐1.395	.153

ALB = albumin, BUN = blood urea nitrogen, CRP = C-reactive protein, Glu = glucose, NLR = neutrophil-to-lymphocyte ratio, TBIL = total bilirubin.

### 3.6. Comparison of the impact of psychological conditions on complications in patients with pancreatitis

The psychological condition of patients with pancreatitis was investigated using the Hospital Anxiety and Depression Scale within 48 hours of admission to the hospital. As shown in Table 6, the anxiety score, depression score, and total social support score of patients with pancreatitis have an impact on the occurrence of complications. The difference possessed SS (*P* < .05).

**Table 6:** Comparison of the impact of psychological conditions on complications in patients with pancreatitis.

Project	No complications (n = 73) *M* (*P*25,*P*75)	Complications present (n = 27) *M* (*P*25,*P*75)	*Z*	*P*
Anxiety (points)	7 (6,9)	11 (8.5,13)	‐7.532	<.001
Depression (points)	4 (2,8)	8 (75,11)	‐7.578	<.001
Social support (points)	36 (30,40)	32 (29,37.5)	‐1.962	.036

### 3.7. Comparison of early nursing care for pancreatitis patients

As shown in Table 7, the difference between patients’ anxiety situation score and patients’ depression situation score before and after the early psychological care of pancreatitis patients was statistically significant for patients before and after care (*P* < .05).

**Table 7:** Comparison of early psychological care for pancreatitis patients.

Project	Before nursing	After care	*Z*	*P*
Anxiety (points)	6 (4,8)	5 (3,6)	‐3.256	<.001
Depression (points)	3 (2,5)	2 (1,3)	‐3.658	<.001
Social support (points)	37 (30,39)	29 (24,36)	‐2.354	.005

### 3.8. Comparison of early care pain in patients with pancreatitis

As shown in Table 8, there was an increase in the number of people who had pain relief before and after care for AP and a decrease in the number of people who had longer pain durations. The median pain scale median pain relief time before care was 4 (2,6), time after care decreased to 3 (1,4), and pain duration decreased from 6 (6,36) to 4 (2,26).

**Table 8:** Comparison of early care pain in patients with pancreatitis.

Project	Classification	Pre-nursing *M* (*P*25, *P*75)	Post-care *M* (*P*25, *P*75)
Pain relief situation	Pain relief within 24 hours [n (%)]	58 (58)	67 (67)
	No pain relief within 24 hours [n (%)]	42 (42)	36 (36)
	Pain relief within 24–48 hours [n (%)]	18 (18)	29 (29)
	No pain relief after 24–48 hours [n (%)]	82 (82)	72 (72)
Analgesic frequency (times)	/	1 (0,2)	1 (0,1)
Total accumulated time of pain during treatment (days)	/	3 (2,5)	2 (1,4)
Pain scale	Duration of mild to moderate pain within 24 hours (h)	11 (11,19)	8 (7,11)
	Severe pain duration within 24 hours (h)	4 (2,6)	3 (1,4)
	24–48 hours of mild to moderate pain duration (h)	5 (5,20)	3 (2,6)
	24–48 hours of severe pain duration (h)	0 (0,1)	0 (0,0)
	48 hours of total pain time (h)	6 (6,36)	4 (2,26)

### 3.9. Analysis of binary logistic regression on the incidence of complications in patients with pancreatitis

As shown in Table 9, binary logistic regression was used to assign values to the inpatient analgesic status, history of hyperlipidemia, age, alcohol consumption, recurrence, history of hypertension, and smoking status of patients with pancreatitis. The study investigated the influencing factors of complications during the admission period of early pancreatitis patients, and assigned values to individual influencing factors as shown in Table 9.

**Table 9:** Binary logistic regression changes in the incidence of complications in patients with pancreatitis.

Argument	Value
Age	<60 years = 0, ≥60 years = 1
Alcohol consumption	Not drinking or abstaining from alcohol = 1, drinking = 0
Smoking situation	Not smoking or having quit smoking = 1, smoking = 0
Is this a relapse	No = 1, Yes = 0
Previous history of hyperlipidemia	No = 1, Yes = 0
Previous history of hypertension	No = 1, Yes = 0
Pain relief within 24 hours of admission	No pain relief = 1, pain relief = 0
Pain relief within 24–48 hours of admission	No pain relief = 1, pain relief = 0
Other variables	Numerical variable

### 3.10. The impact of hospitalization duration on the incidence of complications in patients with pancreatitis

As shown in Table 10, there is a statistically difference (*P* < .05) in the length of hospital stay between patients with pancreatitis and their past history of hypertension, history of hyperlipidemia, current smoking status, and current recurrence of pancreatitis.

**Table 10:** The impact of hospitalization duration on the incidence of complications in patients with pancreatitis.

Project	Classification	Hospitalization time < 7 days (n = 46) [n (%)]	Hospitalization time ≥ 7 days (n = 54) [n (%)]	χ^2^	*P*
Smoking Status	Smoke	16 (16)	14 (14)	4.564	.025
	Not smoking or quitting smoking	30 (30)	40 (40)	/	/
Alcohol consumption status	Drink	15 (36.0)	12 (12)	1.024	.241
	Not drinking or abstaining from alcohol	31 (64.0)	42 (42)	/	/
Is this a recurrence	Yes	11 (11)	22 (22)	4.654	.019
	No	25 (25)	32 (32)	/	/
Hypertension	Yes	22 (22)	23 (23)	3.874	.034
	No	24 (24)	31 (31)	/	/
Hyperlipidemia	Yes	23 (23)	20 (20)	5.732	.012
	No	23 (23)	34 (34)	/	/
The cause of this illness	Biliary	12 (12)	18 (18)	8.546	.054
	High fat content	13 (13)	11 (11)	/	/
	Alcoholic	2 (2)	6 (3.1)	/	/
	Idiopathic	15 (15)	18 (18)	/	/
	Mixed etiology	4 (4)	1 (1)	/	/

### 3.11. Analysis of the impact of etiology and complications on patients with pancreatitis

**Figure 1.**
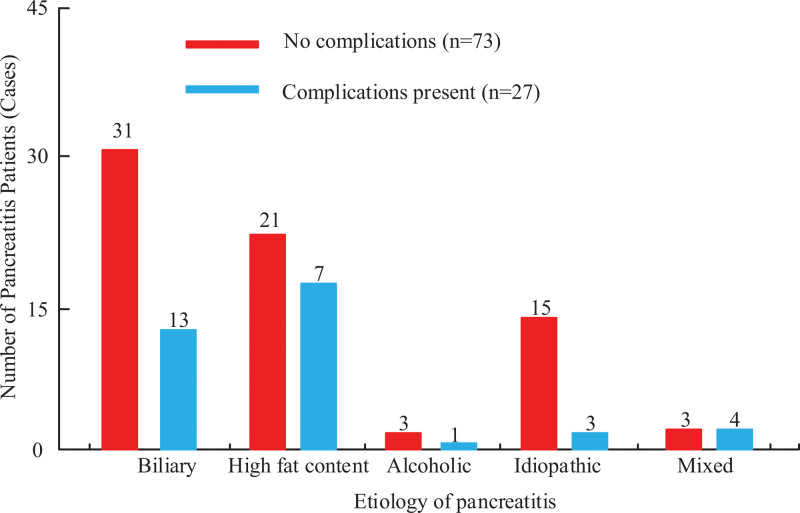
Analysis of the impact of etiology and complications on patients with pancreatitis.

As shown in Figure 1, the analysis of factors influencing the occurrence of complications in patients with pancreatitis is presented. Figure 1 shows that there are 31 patients with biliary disease and no complications, and 13 patients with complications. There were 21 patients with biliary disease and no complications, and 7 patients with complications. There were 3 patients with biliary diseases without complications, and 1 patient with complications. There were 15 patients with biliary disease and no complications, and 3 patients with complications. There were 3 patients with biliary diseases without complications, and 4 patients with complications. There was a significant statistical difference (*P* < .05) between the number of patients with biliary and mixed causes in uncomplicated patients. There was no significant statistical difference (*P* > .05) in the number of patients with alcoholic and idiopathic causes among those with complications.

### 3.12. Analysis of clinical data on complications in patients with pancreatitis

Table 11 shows that pancreatic patients have an impact on the prolongation of hospital stay in AP patients in terms of eating days, BISAP score, time of first defecation, NLR, Glu, CRP, calcium, ALB, and total bilirubin, with SS (*P* < .05).

**Table 11:** Clinical data of patients with pancreatitis and their impact on the factors causing complications.

Project	Hospitalization time < 7 days (n = 46)	Hospitalization time ≥ 7 days (n = 54)	*Z*	*P*
Fasting days (days)	2 (2,4)	6 (5,8)	‐8.548	<.001
BISAP score (points)	1 (0,1)	1 (2,4)	‐9.642	<.001
First bowel movement time (days)	1 (1,3)	2 (4,6)	‐6.125	<.001
NLR (%)	6 (3, 10)	12 (11, 22)	‐4.325	<.001
Glu (mmol/L)	26.78 (2.54, 84.02)	162.46 (89.98, 219.86)	‐7.562	<.001
CRP (mg/L)	1.84 (1.946,25.21)	1.68 (1.76, 2.21)	‐6.485	<.001
BUN (mmol/L)	4.84 (3.87, 6.45)	5.896 (4.51, 9.56)	‐3.792	.01
Ca (mmol/L)	2.13 (2.04,2.19)	1.87 (1.82,2.09)	‐6.723	<.001
ALB (g/L)	43.21 (39.01, 46.12)	33.75 (29.64, 37.89)	‐8.492	<.001
TBIL (μmol/L)	22.12 (13.13, 35.86)	23.76 (18.12, 37.95)	‐1.153	.142
Project	Hospitalization time < 7 days (n = 46)	Hospitalization time ≥ 7 days (n = 54)	*Z*	*P*

ALB = albumin, BUN = blood urea nitrogen, CRP = C-reactive protein, Glu = glucose, NLR = neutrophil-to-lymphocyte ratio, TBIL = total bilirubin.

## 4. Discussion

AP, as an acute digestive system disease, is characterized by a sudden inflammatory response of the pancreas, which may be accompanied by severe local or systemic complications.^[17]^ According to statistics, the incidence of AP has been increasing year by year, becoming one of the common and critical diseases in emergency departments. The clinical manifestations of AP are diverse, including abdominal pain, indigestion, nausea and vomiting, etc. In severe cases, it can lead to multiple organ failure or death.^[18]^ The treatment of AP mainly relies on early diagnosis, effective supportive treatment, and targeted management of the etiology. Early care is particularly important in the treatment of AP, with the aim of controlling disease progression, preventing complications, and improving cure rates.^[19]^ Early care includes but is not limited to pain management, nutritional support, monitoring vital signs, psychological support, and health education. However, due to individual differences in patients, uncertainty in the condition, and limitations in nursing resources, early care for AP faces many challenges in practice.^[20]^

When observing the general baseline data of patients, it was found that 61% of patients were male, and the proportion of patients over 60 years old was as high as 72%. This indicated that the majority of pancreatitis in the emergency department patients are male elderly patients, so it is necessary to strengthen the care and prevention of male elderly patients. At the same time, a higher proportion of patients with high body mass index values indicated that a poorly structured diet can lead to frequent pancreatitis in the emergency department. Patients with high blood pressure, high blood pressure, high blood pressure, and high blood pressure, as well as those who smoke and drink alcohol, were relatively small in the study, indicating that other adverse behaviors have a smaller impact on pancreatitis in the emergency department. The incidence of pain and pain in the early stages of patient admission was as high as 99.85%. Within 24 hours of patient admission, the longest duration of mild to moderate pain was 24 hours, with a median of 11 hours. The longest duration of severe pain was 4 hours, and the longest duration of mild to moderate pain was still 24 hours, with a median of 5 hours. This indicated that the incidence of pain in patients in the study was relatively high, and the time period for nursing care needed to be given special attention due to the different occurrence nodes. In the study of patient complications, 27 patients (27%) experienced local or systemic complications. Among the systemic complications, 30 patients (30%) experienced systemic complications. Sixty-four patients (64%) experienced prolonged hospitalization and 2 patients (2%) died due to worsening of their condition and varying degrees of organ failure. Before and after the care, the pain duration of pancreatitis patients was reduced, and the difference between the patient’s anxiety condition score and the patient’s depression condition score before and after the early psychological care of pancreatitis patients was statistically significant for the patients before and after the care (*P* < .05). When pancreatitis in the emergency department occurs, many physical indicators of the patient will correspondingly increase, causing various inflammatory conditions in the body, which can lead to organ damage or death. Therefore, when caring for the patient, it is necessary to always pay attention to the changes in the patient’s physical indicators to cope with various adverse situations. In patients with AP, there is a statistically SD (*P* < .05) between smoking and a history of fatty liver disease in the occurrence of complications. In the comparison of clinical data and complications in patients with pancreatitis, fasting days, BISAP score, patient’s first bowel movement time, NLR, etc. all have an impact on the occurrence of complications in patients with pancreatitis. The difference possessed SS (*P* < .05). This indicates that improving the gastrointestinal diet and intestinal environment of pancreatic patients can have a positive impact on patients, reduce the occurrence of complications, and shorten their hospital stay. This has a significant guiding role in early care and prognostic interventions for patients. The anxiety score, depression score, and total social support score of patients with pancreatitis have an impact on the occurrence of complications, and the differences are statistically significant (*P* < .05). This indicates that strengthening the psychological construction of patients during their admission period ensures their mental health, which has a good promoting effect on the discharge time and treatment cooperation of patients.

In summary, when patients are admitted for early treatment, it is necessary to establish a pain management system, use analgesics reasonably, pay attention to the patient’s diet and intestinal condition, and reasonably guide the patient’s mental health. It is also necessary to constantly pay attention to the intestinal and experimental indicators of patients, and enhancing their treatment confidence is a good means to ensure and help patients recover quickly. Although this study achieved many results, there are still many shortcomings, such as the inability to determine the causal relationship between current factors and indicators in experimental investigations because it is an investigative analysis. Therefore, more control groups will be added for factor validation analysis in the future. At the same time, there was no specific analysis of the complications of pancreatitis in the emergency department mentioned in the study. Therefore, specific analysis will be conducted on the occurrence and influencing factors of complications in pancreatitis in the emergency department in the future.

## Author contributions

**Conceptualization:** Lejuan Xu, Fengxin Li, Jiehua Wu, Liang He.

**Data curation:** Lejuan Xu, Fengxin Li, Jiehua Wu, Zhe Gao.

**Investigation:** Fengxin Li, Jiehua Wu, Liang He.

**Methodology:** Jiehua Wu, Liang He, Zhe Gao.

**Visualization:** Liang He.

**Writing – original draft:** Lejuan Xu, Fengxin Li.

**Writing – review & editing:** Lejuan Xu, Fengxin Li.
